# *Toxoplasma gondii *infection in humans in China

**DOI:** 10.1186/1756-3305-4-165

**Published:** 2011-08-24

**Authors:** Peng Zhou, Zhaoguo Chen, Hai-Long Li, Haihong Zheng, Shenyi He, Rui-Qing Lin, Xing-Quan Zhu

**Affiliations:** 1 State Key Laboratory of Veterinary Etiological Biology, Key Laboratory of Veterinary Parasitology of Gansu Province, Lanzhou Veterinary Research Institute, Chinese Academy of Agricultural Sciences, Lanzhou, Gansu Province 730046, P R China; 2 Key Laboratory of Animal Parasitology of Ministry of Agriculture, Shanghai Veterinary Research Institute, Chinese Academy of Agricultural Sciences, Shanghai 200241, P R China; 3 Department of Parasitology, College of Veterinary Medicine, South China Agricultural University, Guangzhou, Guangdong Province 510642, P R China; 4 Department of Pig Infectious Diseases, Shanghai Veterinary Research Institute, Chinese Academy of Agricultural Sciences, Shanghai 200241, P R China; 5 Department of Parasitology, Shandong University School of Medicine, Jinan, Shandong Province 250012, P R China; 6 College of Animal Science and Veterinary Medicine, Heilongjiang Bayi Agricultural University, Daqing, Heilongjiang Province 163319, P R China; 7 College of Animal Science and Technology, Yunnan Agricultural University, Kunming, Yunnan Province 650201, P R China

**Keywords:** *Toxoplasma gondii*, Epidemiology, Human, Infection, China

## Abstract

Toxoplasmosis is a zoonotic infection of humans and animals, caused by the opportunistic protozoan *Toxoplasma gondii*, a parasite belonging to the phylum Apicomplexa. Infection in pregnant women may lead to abortion, stillbirth or other serious consequences in newborns. Infection in immunocompromised patients can be fatal if not treated. On average, one third of people are chronically infected worldwide. Although very limited information from China has been published in the English journals, *T. gondii *infection is actually a significant human health problem in China. In the present article, we reviewed the clinical features, transmission, prevalence of *T. gondii *infection in humans in China, and summarized genetic characterizations of reported *T. gondii *isolates. Educating the public about the risks associated with unhealthy food and life style habits, tracking serological examinations to special populations, and measures to strengthen food and occupational safety are discussed.

## Background

Toxoplasmosis, a cosmopolitan disease in humans and most mammals, is caused by the opportunistic protozoan *Toxoplasma gondii *mainly through peroral infections, bloodstream infections and congenital acquired infections. It has been estimated that one third of the world population has been infected [1,2]. In most adults it does not cause serious illness, however, blindness and mental retardation can be caused in congenitally infected children and severe diseases in those with compromised immunity. A recent study indicated that infection with *T. gondii *is associated with abdominal hernia [3].

*Toxoplasma gondii *needs both definitive hosts and intermediate hosts to complete its sexual and the asexual replication phases in life cycle. The sexual phase only occurs in the intestine of the definitive hosts, felids. All the warm-blooded animals, the intermediate hosts, become infected mainly by consuming food or drink contaminated by oocysts evacuated from felids and tissue cysts from other intermediate hosts [4]. Acute infection happens in the initial few days, with the rapidly growing replication of the tachyzoites. Tachyzoites switch to bradyzoites as time goes by and form tissue cysts parasitizing in host cells. It would be lethal in *Toxoplasma *infected immune-compromised patients if bradyzoites revert to tachyzoites. In addition to felids, intermediate hosts carried with tachyzoites or tissue cysts are also responsible for the spread of *T. gondii*. Peroral infection, congenital and blood infection are three major ways for the transmission of this parasite [5].

The first human case of toxoplasmosis in China was report in 1964 in Jiangxi Province [6]. Many human cases were reported in China since the first epidemic survey on toxoplasmosis was carried out in Guangxi Province in 1978 [7]. However, toxoplasmosis cases in China are hardly recognized by western clinicians, for very little information from China was published in English. The rising prevalence of *T. gondii *infection and the increasing clinical cases [8-10] in immunocompromised patients, and patients with congenital toxoplasmosis and psychosis should draw our attention to address toxoplasmosis as a serious public health problem. It is thus timely and appropriate to review the Chinese literature on *Toxoplasma *and toxoplasmosis to gain an improved insight into its epidemiology and socioeconomic impact in China. Most of the data quoted in this review were derived from articles published in Chinese on *T. gondii *infection in humans up to 2011, obtained from The China National Knowledge Infrastructure (CNKI) database via its website http://www.cnki.net, using the keywords "*Toxoplasma*", "Toxoplasmosis" and "Epidemiology". We intend to provide clinical information, to summarize some key aspects regarding transmission and prevalence of human toxoplasmosis, and genetic characterizations of *T. gondii *strains in China, and to conclude by making some suggestions for further research and recommendations for the prevention and control of this important human protozoan disease.

## Epidemiology

Between 2001 and 2004, the national serological survey of 47,444 people in 15 provinces and autonomous regions estimated a mean prevalence of 7.9% by using enzyme-linked immunosorbent assay (ELISA) [11]. Prevalence in these provinces has increased from 5.2% in the first national survey conducted between 1988 and 1992 [12]. Though a recent serological survey carried out in 3281 individuals from the northeast and the south of China showed a 12.3% anti-*T. gondii *IgG positive rate, which indicated the increasing growth in prevalence [13], the prevalence of *T. gondii *in China was still relatively low, comparing with 50-75% seropositive in France, and 20% in UK [14].

Guizhou province and Guangxi province had the highest levels with prevalence of 15.1% and 12.7%, respectively [15]. Prevalence in Miao (25.4%), Buyi (25.3%), Mongol (17.1%) and Zhuang (16.7%) ethnic groups are higher than in other groups of the population [15]. Eating habits play an important role in the parasite infection. People living in southwest of China enjoy eating raw or half-raw meat or animal internal organs in their tradition, such as the 'Sour-meat' (let raw pork or beef ferment and become sour), the 'Shengpi' (is a cooking method which keeps pork and beef half-raw), and even raw pig or cattle liver [16,17]. The Mongol people like to eat their traditional food 'Mongolian Finger Mutton' by hands after contact with animals or raw meat without appropriate washing procedures, because of the water shortage in their living areas, which could lead to *Toxoplasma *infection, in addition to other food-borne diseases such as taeniasis, cysticercosis, trichinelliasis and echinococcosis [15].

## Human toxoplasmosis in mainland China

### Immunocompromised patients with toxoplasmosis

Toxoplasmosis can be life-threatening in immunocompromised patients as a result of reactivation of chronic infection. High seroprevalence of latent *T. gondii *infection has been found among immunocompromised patients. Prevalence of *T. gondii *infection in cancer patients ranged from 24% [18] to 79% [19]. Prevalence of *T. gondii *infection varied with different cancers. Rectal cancer and nasopharyngeal carcinoma sufferers in Changchun city had the highest infection, with prevalence of 63.6% and 46.2%, respectively [18]. However, *T. gondii *infections in patients with breast cancer, hepatocellular carcinoma and gastric carcinoma in Henan Province were much higher than with other cancers, with a prevalence of 78.9%, 77.8% and 61.1%, respectively [19]. The average prevalence of *T. gondii *infection was 15.1% in leukemia patients. Moreover, *T. gondii *prevalence in refractory cases of leukemia patients was up to 35% [20].

Surveys of *T. gondii *infection in the individuals with tuberculosis and hepatitis B showed that the prevalence were 35.3% [21] and 19.3% [22], respectively. Most of the cases belonged to chronic infections. 70% of individuals infected with *T. gondii *and tuberculosis had the experience of intimate contact with animals [21]. Similarly, high prevalence of *T. gondii *infection have been detected among immunocompromised patients especially suffering from malignancy in Egypt and Korea [23,24]. Although there is no direct association between the susceptibility of toxoplasmosis and the immunocompromised state, the high seroprevalence in immunocompromised patients indicates considerable risks, because toxoplasmosis is a consequence of recrudescence of a previously latent infection in most immunocompromised patients, with the chronic parasite infection activation after the immune system was damaged [25].

The first case of AIDS patient died in China was reported in 1991, and *T. gondii *was found in the cerebellum of this patient [26]. In a more recent report, 26% of HIV infected patients in Xinjiang were seropositive with *T. gondii *infection [27].

The CNS is the site most typically affected by *T. gondii *infection. A series of clinical manifestations including mental status changes, seizures, focal motor deficits, cranial nerve disturbances, sensory abnormalities, cerebellar signs, movement disorders, and neuropsychiatric findings have often been found among toxoplasmic encephalitis (TE) cases. The incidence of TE was from 0.6% [28] to 10.5% [9] among the HIV infected patients in Henan and Yunnan Provinces, with all the CD_4_^+ ^T-lymphocyte count below 100 cells per microliter. Toxoplasmic lymphadenitis and retinitis were also found in HIV-infected patients, with the incidence of 6% (2/33) [29] and 5.3% (2/38) [9], respectively.

### Congenital toxoplasmosis

Congenital infection caused by transplacental transmission can lead to a wide variety of manifestations in the fetus and infant including spontaneous abortion, still-birth, a newborn with classic signs of congenial toxoplasmosis such as hydrocephalus or microcephalus, cerebral calcifications and retinochoroiditis [30,31]. There were no national reports about congenital toxoplasmosis on newborns in China. Previous studies focused on certain areas with severe cases. The seroprevalence of *T. gondii *in newborns was between 4.4% to 19.4% in Huizhou city, Shantou city and Dalian city [32-34]. A survey of *T. gondii *infection in 80 puerperas and their new-born babies showed that the seroprevalence were 8.8% and 6.3%, respectively. The vertical transmission was 70% by using ELISA, which was still serious in Xinxiang city of Henan Province [35].

Most infected newborns have no apparent physical abnormalities at birth, but without treatment, most of them may later develop chorioretinitis, neurologic damage or growth can be delayed later in life. The seroprevalence of *T. gondii *infection in disabled children with symptoms of hypophrenia, epilepsy, retinochoroiditis, cardiovascular defects and respiratory system defects were 21.7%, 20%, 26.1%, 25% and 14.3%, respectively [36]. A case-control study in Hainan Province, involving 79 cases of infantile cerebral palsy, revealed that the seroprevalence of *T. gondii *was 41.8%, with significant difference (*P *< 0.001) from the 256 control cases with seroprevalence of 8.6% [37]. A survey of 592 congenital defect infants in Nanjing city, Jiangsu province revealed a 28.13% seroprevalence of *T. gondii *infection, which was significantly higher compared with the normal ones (0.6%) [10].

### Ocular toxoplasmosis

Ocular toxoplasmosis (OT) may induce more than 30% posterior uveitis cases in western populations [38]. Even though both congenital and acquired infection may develop ocular lesions, it has been suggested that 70% of chorioretinal scar formation is of congenital origin [39]. Congenital ocular toxoplasmosis mainly causes congenital malformation, with anophthalmus, congenital aniridia, chorioretinal, congenital cataract, optic neuritis, strabismus, amblyopia, nystagmus, optic atrophy, visual field defects, etc. Ocular lesions originated from acquired infections occur after birth, which resulted in tissue destruction and inflammatory responses [40]. The first case of OT reported in China also was found in the first human toxoplasmosis case in Jiangxi Province in 1964 [6]. The incidence of OT in China is still unclear. The seroprevalence of OT in ophthalmocace patients was up to 38.8% [41]. Central exudative chorioretinitis and uveitis are the most common symptoms in OT cases reported in China [42,43].

### Toxoplasmosis and schizophrenia

Evidence is mounting to link toxoplasmosis with schizophrenia or similar psychiatric disorders. Recent studies revealed that levels of antibodies to *T. gondii *have been found to be increased in individuals with schizophrenia compared to controls with an odds ratio for *Toxoplasma *seropositivity between 2 and 4.4 [44-47]. In prospective studies, an increase in IgG antibodies to *T. gondii *has been found in mothers of infants who later develop schizophrenia [48].

Many reports revealed that *Toxoplasma *might represent a major pathogen in some cases of psychosis. It has been proven that the parasite infection could increase the dopamine level in mice brains [49]. Dopamine plays a key role in psychosis cases such as schizophrenia, and bipolar disorder [50-52]. The seroprevalence of *T. gondii *infection in schizophrenic patients ranged from 1% to 28.7% in China [11,53]. A report of 67 cases of childhood schizophrenia between 6~17 years old revealed a *Toxoplasma*-IgG positive rate of 85.7% [54]. Another survey on *Toxoplasma *infection in 600 cases of the first episode of schizophrenia revealed that the *Toxoplasma*-IgG positive rate was 13.7% [55]. The prevalence of *Toxoplasma *IgG in mothers of 252 was 34.8% [55]. The alterations of behavior and psychomotor skills in schizophrenia patients have also been found to be associated with the *T. gondii *infection [56]. Compared with the seronegative ones, clinical manifestations of excitation, hostility, mannerisms and posturing, disturbance of volition, poor impulse control and anger, difficult to delay fulfilling request and suspiciousness in the seropositive patients were statistically different [56]. The schizophrenic patients living in rural area have a higher infection rate than these lived in cities, with prevalence of 28.6% and 6%, respectively [57].

### Poor obstetric outcomes, sterility and toxoplasmosis

There is as yet no direct evidence showing the association between toxoplasmosis and sterility in women. Nevertheless, some studies have demonstrated that *T. gondii *infection could cause reproductive failure in mice, which was due to an acquired hypogonadotropic hypogonadism secondary to hypothalamic dysfunction, exhibiting histopathological changes with estrus cycling cessation, impaired folliculogenesis and few corpora lutea [58,59]. It seems that *T. gondii *infection in pregnant women may cause poor obstetric outcomes such as spontaneous abortion, hydatidiform mole, still-born, teras and sterility. Women who had a poor obstetric outcome history had a seroprevalence of 14.2% [60] to 33.9% [61] which was much higher than that of the normal pregnancy in China. A survey of *T. gondii *infection in 68 cases of oviducal sterility revealed a prevalence of 44.1%, which was significantly different from that in normal pregnant women (3.3%) [62], indicating that *Toxoplasma *infection could result in oviducal sterility.

*T. gondii *infection is also found to be related with the male sterility. Recent zoopery studies revealed that the reproductive parameters including sperm motility and sperm concentration were significantly decreased in *T. gondii *infected rats, and a marked increase in sperm abnormalities was also found in these infected male rats [63]. Similar results were also observed in male mice experimentally infected with *T. gondii *[64]. Zhou (2002) [65] found that *Toxoplasma *infection in infertile human couples was higher than that in fertile couples, possibly related to the antisperm antibodies which were higher in *Toxoplasma*-infected couples. A recent investigation of *T. gondii *infection in 100 men with sterility revealed that 16% of them were IgM-positive and 13% were CAg-positive, significantly higher than in healthy men [66]. The seroprevalence of *Toxoplasma *infection in male sterility cases were 19.8% in Luoyang, Henan province [67], to 22.8% in Yan'an, Shaanxi province [68], significantly higher than in the healthy men. Based on a number of relevant studies and investigations in China, it is concluded that *T. gondii *infection may result in male sterility [69].

### Transmission of toxoplasmosis

#### Transmission by contact with animals

Some occupations required people to have contact with animals and meats and these frequently possess higher risk of infection with the parasite, such as dairy workers, slaughterhouse workers, veterinarians, meat-processing workers, meat sellers and cooks. Selected serological surveys of *T. gondii *infection in different occupations in China are summarized in Table 1.

**Table 1:** Serological surveys of *Toxoplasma gondii *infection in people of different occupations in China

Occupations	Year	Area and Province	**Serologic test**^**a**^	No. tested	Positive	Reference
Slaughterhouse Workers	2003	Haerbin, Heilongjiang	ELISA	86	25.6%	[70]
	2005	Anshun, Guizhou	ELISA	100	45.0%	[71]
Dairy Workers	2004	Guangzhou, Guangdong	ELISA	459	10.2%	[72]
	2004	Huadu, Guangdong	ELISA	25	24.0%	[73]
Veterinarians	2002	Lanzhou, Gansu	ELISA	24	12.5%	[74]
	2005	Anshun, Guizhou	ELISA	100	26.0%	[71]
Meat-processing workers	2004	Huadu, Guangdong	ELISA	131	13.7%	[73]
	2005	Anshun, Guizhou	ELISA	100	21.0%	[71]
Cooks	1996-1999	Wuxi, Jiangsu	ELISA	627	29.7%	[75]
	2004	Huadu, Guangdong	ELISA	150	10.0%	[73]
Animal Breeder	2002	Lanzhou, Gansu	ELISA	25	20.0%	[74]
	2005	Anshun, Guizhou	ELISA	100	12.0%	[71]

^a ^ELISA: enzyme-linked immunosorbent assay

These data suggest that the oral route is the major route of infection. Pork is one of the most popular meats in China. Seroprevalence of *T. gondii *in pigs is high in some Chinese provinces, for instance 16.9% in Yunan province [76], 27% in Guangdong province [77] and 53.4% in Zhejiang province [78], which are higher than that in USA (2.7%, [79]), Germany (4.1%, [80]) and Mexico (12.7%, [81]). There is still no meat inspection for *T. gondii *contamination in meat before it is sold for human consumption, nor any strict performance standards for processing meat and animal products, which gives a high risk of infection for these workers. Similar high prevalences of *T. gondii *infection have also been found in butchers in Finland [82], Egypt [83] and Brazil [84]. A latest Mexican report proposed that occupational exposure to raw meat has no correlationship with seropositivity of *T. gondii *infection [85]. The reason may be associated with good sanitary conditions in the slaughterhouses and powerful protective facilities to the workers. However, in our investigations of *T. gondii *infection in slaughter pigs in several provinces in China, we found that butchers do not always wear gloves during work because of the hot and humid environment around the slaughterhouses, which may increase the risk for *T. gondii *infection when handling raw meat.

Herdsmen possessed a higher *T. gondii *prevalence in the west China, such as Xinjiang and Inner Mongolia due to inadequate sanitary conditions and the traditional eating habits mentioned above, with seroprevalence ranged from 6.7% [86] to 18.5% [87]. People owning dogs and cats as pets also processed a high risk of infection, with the prevalence ranged from 5.3% [88] to 34.8% [74].

#### Transmission by blood transfusion

*T. gondii *can also be transmitted via blood or leucocytes from infected donors [89]. The seroprevalence of *T. gondii *in blood donors in China varied between 0.4% [90] to 20.2% [91], which were lower than in Egypt (59.6%) [92] and Malaysia (28.1%) [93]. Peasant blood donors had the highest seroprevalence (28.6%), which may be related to the living habits and chances of contacting with animals [91]. Therefore, it is very urgent for a hospital to check all the blood from blood donors. It would be lethal to transmit the parasite from immunocompetent donors to immunocompromised recipients during surgery.

Another pattern of *T. gondii *transmission by blood is though needles among intravenous drug users (IVDU). The prevalence of *T. gondii *in IVDU ranged from 17.3% [94] to 21.8% [95], which were significantly higher (*P *< 0.01) than the prevalence (7.8%) in those who took drugs by the non-intravenous route [95]. Drug addiction history and HIV infection was associated with the susceptibility of *Toxoplasma *in IVDU. IVDU with 5 years or more addiction history possessed higher seroprevalence compared with those less than 5 years, with seropositivity of 21.8% and 8%, respectively [94]. *Toxoplasma *prevalence in drug users with HIV was significantly higher than in those with no HIV infection, being 35.8% (29/81) and 5%, respectively [94].

#### Congenital transmission

When a woman ingests oocysts or cysts of *T. gondii *for the first time during gestation [2], tachyzoites are disseminated through the body by blood. The fetus becomes infected by the entry of *T. gondii *to the fetal circulation though the placenta. Maternal acquisition of *T. gondii *before pregnancy poses a rare risk to the fetus except the infection in immunocompromised women [96]. The risk of congenital infection increases during pregnancy. The acquisition within the first pregnancy trimester has the lowest risk (10-15%) of congenital infection, whereas risks of transmission are much higher during the third trimester [2]. Fortunately, the later in pregnancy that congenital infection occurs, the less severe the consequence is to the fetus.

Since more than 90% of chronic toxoplasmosis infections are asymptomatic, primary prevention is the best way to lower the risk of congenital infection. The detection and treatment of *T. gondii *in infected pregnant women would be an efficient way of attempting to reduce congenital transmission. In China, surveys of *Toxoplasma *infection in women were often been carried out before or at the third month of pregnancy. Selected serological surveys in women during pregnancy in different areas in China during 1994 to 2008 are summarized in Table 2. The prevalence of *T. gondii *infection in pregnant women ranged from 0.5% [106] to 25.5% [104] in China, which were lower than those reported in Austra (35%) [113], France (43.8%) [114] and Brazil (53.0%) [115]. The cultural behaviors and living conditions play an important part for the parasite infection in pregnant women. Those pregnant women who had a history of contact with animals and had habits of consuming undercooked meat or some other raw foods such as un-pasteurized milk or raw eggs, possessed a higher risk for infection.

**Table 2:** Serological surveys of *Toxoplasma gondii *infection in pregnant women during 1996 to 2008 in China

Year	Age group	Area and Province	**Serologic test**^**a**^	No. tested	Positive	Reference
1996	NA	Nanning, Guangxi	IHA	1495	7.0%	[97]
1999-2002	NA	Huainan, Anhui	ELISA	228	10.1%	[98]
2003	20-39	Haerbin, Heilongjiang	ELISA	2184	2.2%	[99]
2003	NA	Nanchang, Jiangxi	ELISA	298	6.0%	[100]
2000-2004	21-37	Chongqing	ELISA	1820	0.8%	[101]
2002-2003	NA	Hongkou, Shanghai	ELISA	1075	3.3%	[102]
2003-2004	23-45	Quanzhou, Fujian	ELISA	550	2.7%	[103]
2000-2005	19-44	Guangzhou, Guangdong	ELISA	1332	25.5%	[104]
2005	20-35	Jilin, Jilin	ELISA	196	9.7%	[105]
2003-2006	NA	Wenling, Zhejiang	IBT	2425	0.5%	[106]
2004-2006	NA	Baoding, Hebei	ELISA	3500	3.6%	[107]
2005-2006	NA	Guizhou	ELISA	769	16.5%	[108]
2001-2007	NA	Wuhan, Hubei	ELISA	18127	13.2%	[109]
2007-2008	NA	Kunshan, Jiangsu	ELISA	1491	7.9%	[110]
2008	21-41	Linqing, Shandong	PM	3559	5.0%	[111]
2008	18-45	Tongliao, Inner Mongolia	ELISA	172	15.1%	[112]

NA: data were not available.

^a^ELISA: enzyme-linked immunosorbent assay, IHA: indirect hemagglutination test, IBT: immunoblotting test, PM: Protein microarray.

#### Genetic characterization of *T. gondii *strains

The information on genetic characterization of *T. gondii *strains prevailing in China is limited even though there have been many reports of seroprevalence of *T. gondii *infection in humans and animals published locally in the Chinese language. But, only a small percentage of exposed humans or animals develop clinical toxoplasmosis, which increases the difficulty for parasite isolation. Based on 10 PCR-RFLP markers, the genetic variability of *T. gondii *isolates from China has been revealed gradually. A total of 5 genotypes were identified, indicating limited diversity of the parasite in China, which is in sharp contrast to South America where a variety of parasite lineages are transmitted in the environment [116]. Clonal Type I lineages were identified from human patients and pigs in China. The unique genotype in cats in Guangzhou city [117], has also been identified from human patients and pigs [116]. It is the major lineage prevalent in Mainland China, which has also been identified from Colombia, Sri-Lanka, Vietnam, and the USA, indicating that it is widespread in Asia, and South and North America [118].

## Conclusion

### Suggested control strategies and measures

The control of human toxoplasmosis is dependent on the reduction or elimination of the transmission of the parasite. However, the effective control of toxoplasmosis is still a difficult task in China. As the oral route is the major method of infection, efforts should be undertaken to guarantee the successful implementation of food safety regulations and regulatory control procedures. The Hazard Analysis and Critical Control Points (HACCP) principles [119], recommended by the Food and Agriculture Organization and WHO to be applied to control food-borne parasite infections in humans, should be evaluated by relevant Chinese authorities by relating to the actual conditions in China. Promoting educational programs directed at reducing environmental contamination with *T. gondii *would eventually reduce the cost of treating humans for clinical toxoplasmosis. There should also be educational programs to guide people to change their habit of consuming uncooked meat and unboiled water, for oocysts can survive for up to 3 years and be transmitted by water through direct drinking [120]. Rules for inspecting meat for *T. gondii *contamination before being sold should be set up and supported by governments.

Detection of antibodies is very important for pregnant women and women of child-bearing age. This is an effective way to find the infection, and then to provide treatment. It is also an efficient way to stop congenital toxoplasmosis in newborns. Good animal husbandry practice and animal welfare should be set up and popularized for food-producing animals, which may also decrease the risk of human infection. If such strategies and measures can be implemented, it should be possible to effectively control, or at least substantially reduce, the prevalence and intensity of human and animal infections with *T. gondii *in China.

## Competing interests

The authors declare that they have no competing interests.

## Authors' contributions

XQZ and PZ conceived and designed the review, and critically revised the manuscript. PZ drafted the manuscript. ZC, HLL, HZ, SH and RQL contributed to drafting the manuscript. All authors read and approved the final manuscript.
